# The RNA Demethyltransferase FTO Regulates Ferroptosis in Major Depressive Disorder

**DOI:** 10.3390/ijms26031075

**Published:** 2025-01-26

**Authors:** Kexin Meng, Zijing Liu, Yuesong Yu, Erning Zhang, Xiaolin Yu, Peixin Meng, Jianbo Xiu

**Affiliations:** 1 State Key Laboratory of Common Mechanism Research for Major Diseases, Institute of Basic Medical Sciences Chinese Academy of Medical Sciences, School of Basic Medicine Peking Union Medical College, Beijing 100005, China; mkx@ibms.pumc.edu.cn (K.M.); cynthia_lzj@163.com (Z.L.); syag777@163.com (Y.Y.); zen17@tsinghua.org.cn (E.Z.); yxl13953187676@163.com (X.Y.); 15648161031@163.com (P.M.); 2 Neuroscience Center, Chinese Academy of Medical Sciences, Beijing 100005, China; 3 State Key Laboratory of Complex, Severe, and Rare Diseases, Beijing 100005, China

**Keywords:** major depressive disorder, RNA methylation, ferroptosis

## Abstract

Major depressive disorder (MDD) is a widespread and severe mental health condition characterized by persistent low mood and loss of interest. Emerging evidence suggests that ferroptosis, an iron-dependent form of cell death, and epigenetic dysregulation contribute to the pathogenesis of MDD. This study investigates the role of RNA demethylase FTO and autophagy regulator BECN1 in ferroptosis and their regulation by the active compound ginsenoside Rb1 (GRb1) as a potential antidepressant strategy. Hippocampal tissues from postmortem MDD patient brains and mice with chronic restraint stress (CRS)-induced depression were analyzed. Ferroptosis was evaluated by analyzing the levels of markers such as glutathione (GSH) and malondialdehyde (MDA). GRb1 was administered to CRS model mice by gavage to explore its effects on ferroptosis-related pathways. The results showed that FTO and BECN1 expression was reduced in the hippocampal tissues of MDD patients and CRS model mice, promoting ferroptosis via disruption of the antioxidant system. Moreover, GRb1 treatment increased FTO and BECN1 expression, modulated m6A methylation, restored the antioxidant balance, and inhibited ferroptosis in CRS model mice. These findings reveal a novel epigenetic mechanism of ferroptosis in MDD and highlight GRb1 as a promising agent for treating depression through the targeting of ferroptosis pathways.

## 1. Introduction

Major depressive disorder (MDD) is a mental health condition that is characterized by persistent low mood and a loss of interest in daily activities [1]. Both genetic and environmental factors play roles in the development of MDD via complex interactions known as epigenetics. Numerous studies have shown that DNA methylation, histone modifications, and miRNA are involved in the onset of depression. DNA hypermethylation of BDNF and NR3C1 has been shown to significantly increase the risk of depression [2]. In the hippocampus of stress model mice, the expression levels of HDAC4 [3] and HDAC5 [4] were significantly elevated, which is consistent with findings in human peripheral leukocytes [5]. Moreover, microRNAs such as miR-199a-5p [6], miR-139-5p [7], and miR-221-3p [8] have been confirmed to play critical roles in various pathophysiological mechanisms of depression within the hippocampus. But research on the role of RNA methylation remains limited [9]. In a previous study, we reported that the FTO gene influences depressive-like behaviors through its RNA demethylase activity, which affects the FTO-ADRB2-SIRT1 signaling pathway [10]. However, further investigations are needed to reveal additional molecular mechanisms underlying MDD, which could inform the development of more effective treatment strategies.

Ferroptosis is a recently identified form of programmed cell death that is distinct from traditional pathways, and it is driven by iron dependency and the failure of GSH-dependent antioxidant defenses [11]. Emerging evidence suggests that various pathways, including epigenetic mechanisms, regulate ferroptosis [12]. Although there are numerous reports that epigenetic regulation of ferroptosis affects cancer development [13], its role of epigenetics in regulating ferroptosis in MDD remains unexplored.

Drug treatments can alleviate depressive symptoms to some extent; however, the therapeutic mechanisms of these drugs remain poorly understood, and their long-term clinical use is limited by varying degrees of toxicity and side effects [14]. Traditional Chinese medicine (TCM), which is a key element in traditional healthcare systems, is widely employed to treat mental disorders. Among TCM treatments, ginseng is widely recognized for its ability to support nervous system function [15] and improve mental health [16]. One of the primary active compounds of ginseng, namely, ginsenoside Rb1 (GRb1), has a range of beneficial effects, including antioxidant [17] and anti-apoptotic effects [18], regulation of inflammatory responses [19], and enhancement of neuroplasticity [20]. Moreover, GRb1 has demonstrated significant antidepressant effects in rodent models of depression [21]. Interestingly, recent studies suggest that GRb1 can induce ferroptosis in hepatic stellate cells, thereby reducing liver fibrosis [22]. However, the potential role of ferroptosis in the antidepressant mechanisms remains unexplored.

In this study, we discovered that FTO modulates BECN1 through its RNA demethyltransferase activity in the mouse hippocampus. BECN1, in turn, induces ferroptosis via the Xc^−^-GSH-GPX4 pathway, contributing to the depressive-like behaviors of mice. Notably, treatment with GRb1 inhibited ferroptosis through the Xc^−^-GSH-GPX4 pathway, thereby exerting significant antidepressant effects.

## 2. Results

### 2.1. Dysregulation of BECN1 and m6A Methylation in MDD Hippocampal Tissues of Individuals with MDD

**Figure 1:**
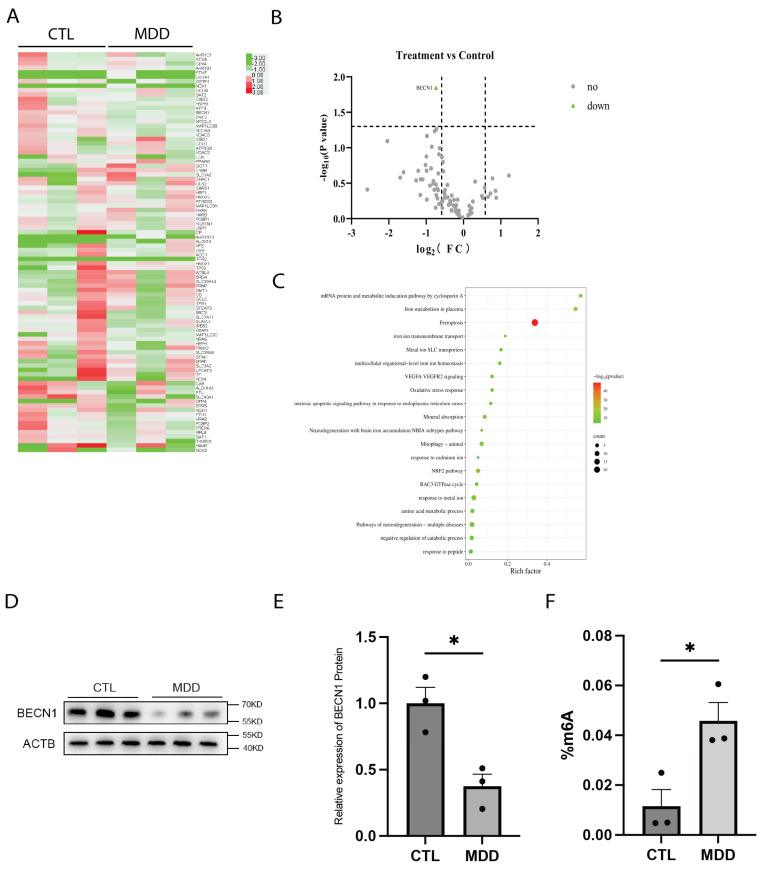
Reduced BECN1 expression in the hippocampus of MDD patients. (**A**–**C**) PCR array analysis heatmap, volcano plot, and KEGG pathway enrichment of ferroptosis-related genes in the hippocampus of MDD patients. *n* = 3 per group. (**D**,**E**) Protein expression of BECN1 in the hippocampal tissues of MDD patients. *n* = 3 per group. *p* = 0.0144. (**F**) m6A levels in the hippocampal tissues of MDD patients. *n* = 3 per group. *p* = 0.0263. * *p*  <  0.05. Two-tailed student’s *t*-test. Error bars represent s.e.m.

To investigate whether ferroptosis contributes to the pathogenesis of MDD, we performed RNA array analysis on RNA that was extracted from the hippocampal tissues of postmortem brains from individuals diagnosed with MDD. Fold-change analysis identified 35 downregulated genes and 5 upregulated genes. Among these genes, only BECN1 exhibited a statistically significant change in expression, as revealed by a volcano plot analysis (Figure 1A–C). BECN1, a key regulator of autophagy, has been reported to participate in ferroptosis through both autophagy-dependent and autophagy-independent mechanisms [23]. We further analyzed BECN1 protein expression in the hippocampal tissues of postmortem brains of individuals with MDD, and we found that BECN1 protein levels were significantly lower than those in hippocampal tissues from control subjects (Figure 1D,E). Our previous study found that the levels of RNA methylation-related enzymes, including the demethylase FTO, in the peripheral blood of MDD patients changed significantly [10]. Therefore, we measured the overall level of m6A in the hippocampal tissues of postmortem brains of MDD patients. The results revealed that the m6A levels were significantly greater in the hippocampal tissues of postmortem brains of MDD patients compared to those of normal controls (Figure 1F).

### 2.2. FTO Regulates BECN1 Expression Through Its m6A Demethyltransferase Activity

**Figure 2:**
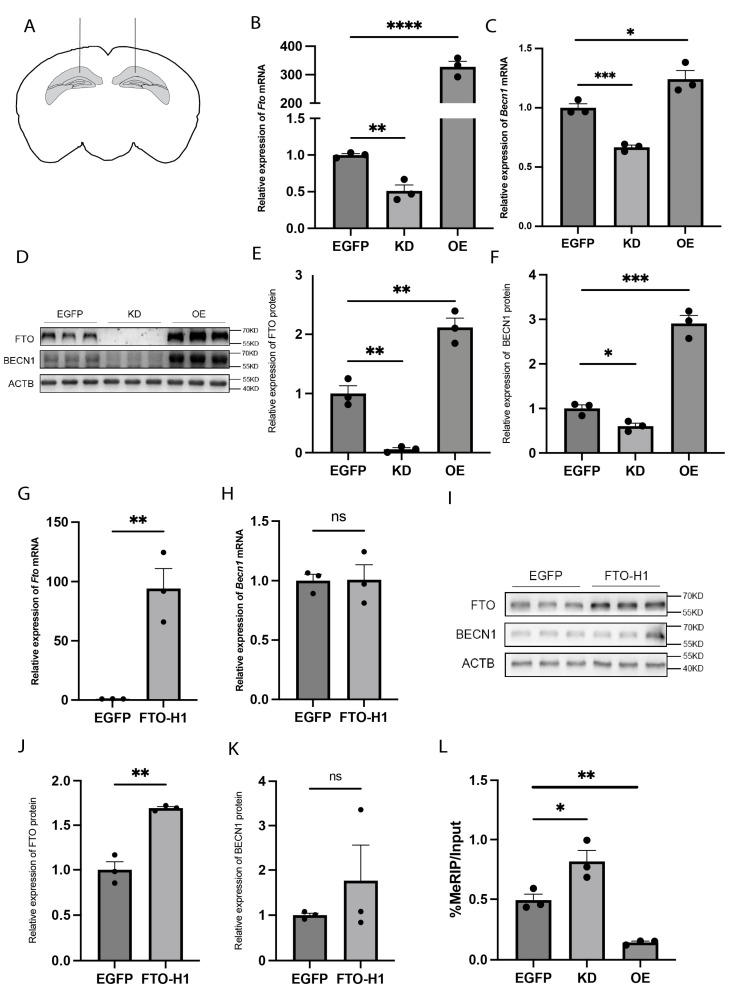
The demethyltransferase FTO regulates BECN1 expression in the hippocampus. (**A**) Schematic diagram of rAAV microinjection. (**B**) Fto mRNA expression levels in the hippocampus of FTO-KD and FTO-OE mice. *n* = 3 per group. EGFP vs. KD, *p* = 0.0044. EGFP vs. OE, *p* < 0.0001. (**C**) Becn1 mRNA expression levels in hippocampus of KD mice and OE mice. *n* = 3 per group. EGFP vs. KD, *p* = 0.0010. EGFP vs. OE, *p* = 0.0379. (**D**–**F**) FTO and BECN1 protein expression levels in hippocampus of KD mice and OE mice. *n* = 3 per group. (**E**) FTO protein expression levels. EGFP vs. KD, *p* = 0.0021. EGFP vs. OE, *p* = 0.0055. (**F**) BECN1 protein expression levels. EGFP vs. KD, *p* = 0.0191. EGFP vs. OE, *p* = 0.0006. (**G**) Fto mRNA expression levels in hippocampus of mice expressing the enzymatically inactive FTO mutant (FTO-H1). *n* = 3 per group. EGFP vs. FTO-H1, *p* = 0.0053. (**H**) Becn1 mRNA expression levels in hippocampus of FTO-H1 mice. *n* = 3 per group. EGFP vs. FTO-H1, *p* = 0.9539. (**I**–**K**) FTO and BECN1 protein expression levels in hippocampus of FTO-H1 mice. *n* = 3 per group. (**J**) FTO protein expression levels. EGFP vs. FTO-H1, *p* = 0.0017. (**K**) BECN1 protein expression levels. EGFP vs. FTO-H1, *p* = 0.3973. (**L**) m6A modification levels of Becn1 mRNA in the hippocampus of KD and OE mice. *n* = 3 per group. EGFP vs. KD, *p* = 0.0372. EGFP vs. OE, *p* = 0.0021. ns *p* > 0.05, * *p*  <  0.05, ** *p*  <  0.01, *** *p*  <  0.001, **** *p* < 0.0001. Two-tailed student’s *t*-test. Error bars represent s.e.m.

To investigate whether FTO regulates BECN1 expression, we used recombinant adeno-associated virus (rAAV) to knock down (KD) or overexpress (OE) FTO in the hippocampus of wild-type C57BL/6 mice (Figure 2A). Four weeks after bilateral stereotaxic injection of the rAAV into the hippocampus of the mice, FTO mRNA and protein expression reached the expected levels in both the FTO-KD and FTO-OE mice (Figure 2B,D,E). Then, we examined the impact of these changes in FTO expression on BECN1 mRNA and protein levels. In FTO-OE mice, the BECN1 mRNA and protein levels were significantly increased, whereas in FTO-KD mice, these levels significantly decreased (Figure 2C,D,F). We then used a plasmid carrying a mutant FTO sequence in which the iron ligand was mutated, resulting in the inactivation of the enzyme activity of FTO [24]. We packaged it into recombinant rAAV and injected it stereotaxically into the mouse hippocampus. In the mice injected with this mutant virus, FTO mRNA and protein levels increased significantly, but BECN1 mRNA and protein levels remained unchanged (Figure 2G–K). To further assess the role of m6A modification, we conducted MeRIP-qPCR to assess the m6A levels of Becn1 mRNA, a method used to measure m6A modification on mRNA. The results revealed that FTO-KD in the hippocampus led to hypermethylation of Becn1 mRNA, whereas FTO-OE caused hypomethylation (Figure 2L). These findings confirm that FTO regulates BECN1 expression through its m6A demethyltransferase activity.

### 2.3. FTO Regulates Ferroptosis via BECN1/xCT

**Figure 3:**
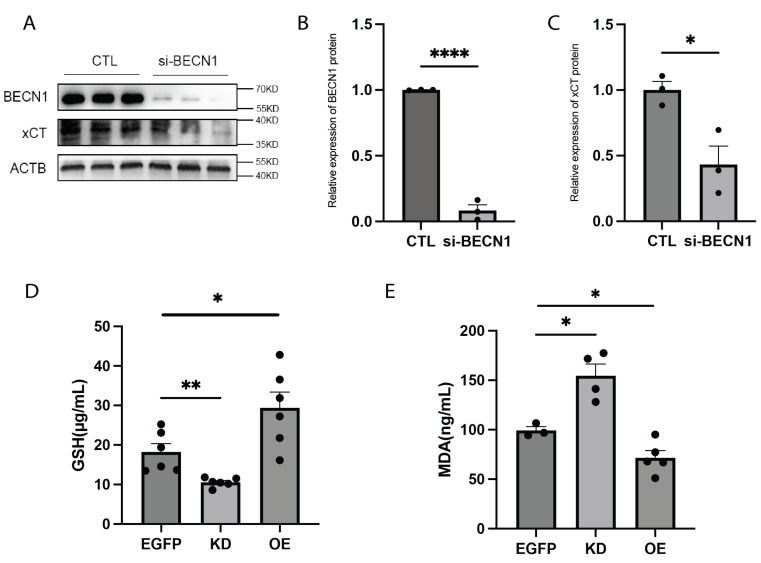
FTO affects cell ferroptosis by regulating BECN1. (**A**–**C**) Protein expression levels of BECN1 and xCT after BECN1 knockdown in 293T cells. *n* = 3 per group. (**B**) Protein expression levels of BECN1. *p* < 0.0001. (**C**) Protein expression levels of xCT. *p* = 0.0215. (**D**) GSH levels in the hippocampus of KD mice and OE mice. *n* = 6 per group. EGFP vs. KD, *p* = 0.0048. EGFP vs. OE, *p* = 0.0324. (**E**) MDA levels in the hippocampus of KD mice and OE mice. EGFP, *n* = 3. KD, *n* = 4. OE, *n* = 5. EGFP vs. KD, *p* = 0.0121. EGFP vs. OE, *p* = 0.0323.* *p*  <  0.05, ** *p*  <  0.01, **** *p* < 0.0001. Two-tailed student’s *t*-test. Error bars represent s.e.m.

The cystine transporter solute carrier family 7 member 11 (SLC7A11), a core component of the cystine/glutamate antiporter (xCT), plays a crucial role in maintaining the intracellular reducing environment by importing cystine to reduce oxidative stress and inhibit ferroptosis. Cystine is converted into cysteine, which is the primary substrate for glutathione (GSH) synthesis. GSH reduces toxic lipid peroxides to nontoxic alcohols through the action of glutathione peroxidase 4 (GPX4) [25]. The xCT-GSH-GPX4 pathway is a well-established regulatory mechanism of ferroptosis [26]. Previous studies have shown that BECN1 influences ferroptosis through the xCT/GPX4 pathway in various types of cancer cells [23]. To investigate the regulatory role of BECN1 in this pathway, we knocked down BECN1 in 293T cells. Protein analysis confirmed the successful KD of BECN1, revealing a significant reduction in xCT protein levels (Figure 3A–C). Moreover, lipid peroxidation is an important feature of ferroptotic cell death, and malondialdehyde (MDA) is the main product of lipid peroxidation. Therefore, the depletion of glutathione (GSH) and the accumulation of MDA are essential hallmarks of ferroptotic cell death. In FTO-KD mice, hippocampal GSH levels were reduced, and MDA levels were increased, indicative of enhanced ferroptosis. Conversely, in FTO-OE mice, GSH levels were increased (Figure 3D), and MDA levels decreased (Figure 3E), suggesting ferroptosis inhibition. These findings indicate that FTO regulates the m6A modification of Becn1 mRNA, which in turn influences the xCT-GSH-GPX4 pathway and modulates ferroptosis.

### 2.4. Ferroptosis Is Observed in the Hippocampus of Mice Exposed to Chronic Restraint Stress (CRS)

**Figure 4:**
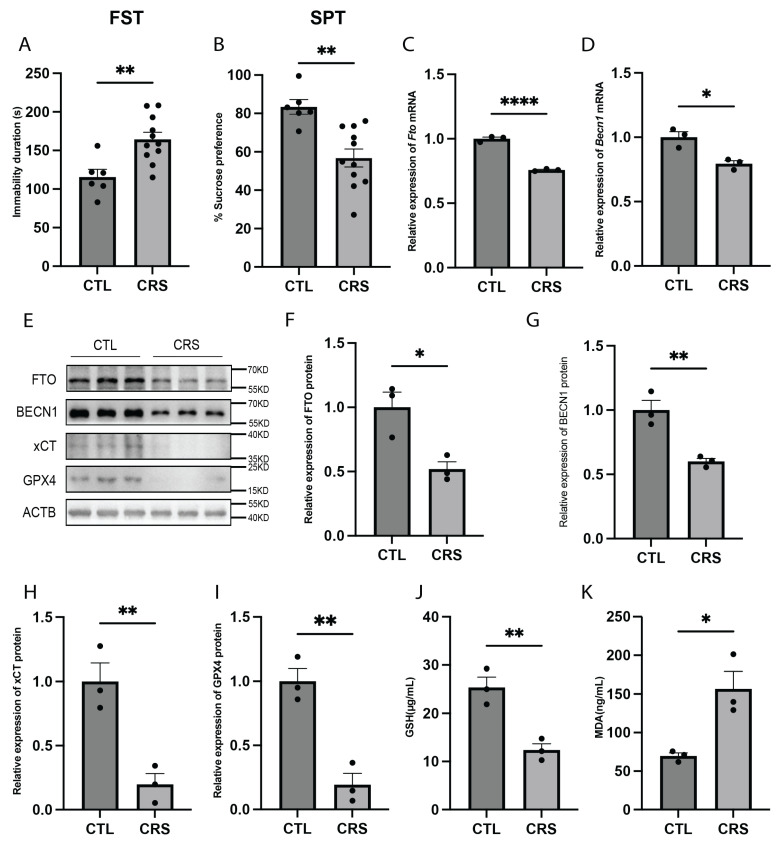
Increased hippocampal ferroptosis in the CRS mouse model. (**A**) FST results of CRS model mice. CTL, *n* = 6. CRS, *n* = 11. *p* = 0.0040. (**B**) SPT results of CRS model mice. CTL, *n* = 6. CRS, *n* = 11. *p* = 0.0018. (**C**) Fto mRNA expression levels in the hippocampus of CRS mice. *n* = 3 per group. *p* < 0.0001. (**D**) Becn1 mRNA expression levels in the hippocampus of CRS mice. *n* = 3 per group. *p* = 0.0138. (**E**–**I**) Protein expression levels of FTO, BECN1, xCT, and GPX4 in hippocampus of CRS mice. *n* = 3 per group. (**F**) FTO protein expression levels. *p* = 0.0212. (**G**) BECN1 protein expression levels. *p* = 0.0074. (**H**) xCT protein expression levels. *p* = 0.0083. (**I**) GPX4 protein expression levels. *p* = 0.0037. (**J**) GSH levels in the hippocampus of CRS mice. *n* = 3 per group. *p* = 0.0065. (**K**) MDA levels in the hippocampus of CRS mice. *n* = 3 per group. *p* = 0.0188. * *p*  <  0.05, ** *p*  <  0.01, **** *p*  <  0.0001 Two-tailed student’s *t*-test. Error bars represent s.e.m.

We established a CRS mouse model, a widely used preclinical model for depression research [27], and confirmed its successful establishment through the forced swimming test (FST) and sucrose preference test (SPT). The results of both tests demonstrated that CRS mice exhibited depressive-like behaviors (Figure 4A,B). Using real-time quantitative PCR (RT-qPCR), we found that FTO and BECN1 expression levels were significantly reduced in the hippocampus of CRS model mice (Figure 4C,D). Western blot analysis further revealed a significant reduction in the protein expression levels of FTO, BECN1, xCT, and GPX4 (Figure 4E–I). Additionally, we measured glutathione (GSH) and malondialdehyde (MDA) levels in the hippocampus. Compared with normal mice, CRS model mice exhibited significantly lower GSH levels and significantly higher MDA levels, indicating the occurrence of oxidative stress and lipid peroxidation (Figure 4J,K). Collectively, these findings suggest that ferroptosis occurs in the hippocampi of CRS model mice and may contribute to the depressive-like behaviors observed in these mice.

### 2.5. GRb1 Exerts Antidepressant Effects by Inhibiting Ferroptosis in CRS Model Mice

**Figure 5:**
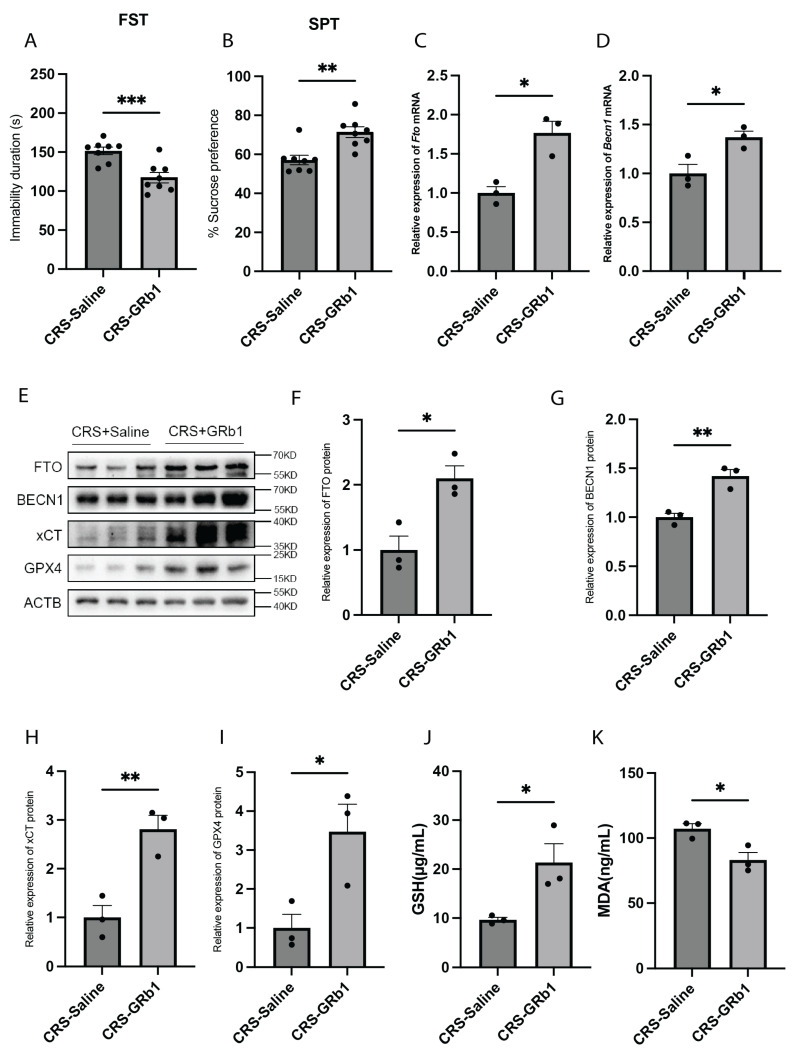
GRb1 can exert antidepressant effects through FTO/BECN1/xCT/GPX4. (**A**) FST results of CRS model mice after GRb1 treatment. *n* = 8 per group. *p* = 0.0009. (**B**) FST results of CRS model mice after GRb1 treatment. *n* = 8 per group. *p* = 0.0016. (**C**) Fto mRNA expression levels in the hippocampus of CRS model mice after GRb1 treatment. *n* = 3 per group. *p* = 0.0107. (**D**) Becn1 mRNA expression levels in the hippocampus of CRS model mice after GRb1 treatment. *n* = 3 per group. *p* = 0.0295. (**E**–**I**) FTO, BECN1, xCT, and GPX4 protein expression levels in the hippocampus of CRS model mice after GRb1 treatment. *n* = 3 per group. (**F**) FTO protein expression levels. *p* = 0.0190. (**G**) BECN1 protein expression levels. *p* = 0.0057. (**H**) xCT protein expression levels. *p* = 0.0084. (**I**), GPX4 protein expression levels. *p* = 0.0348. (**J**) GSH levels in the hippocampus of CRS model mice after GRb1 treatment. *n* = 3 per group. *p*= 0.0386. (**K**) MDA levels in the hippocampus of CRS model mice after GRb1 treatment. *n* = 3 per group. *p* = 0.0252. * *p*  <  0.05, ** *p*  <  0.01, *** *p*  <  0.001. Two-tailed student’s *t*-test. Error bars represent s.e.m.

GRb1, a primary active component of ginseng, has been demonstrated to have potential in treating depression [20,21,28]. Recent research revealed that GRb1 can inactivate hepatic stellate cells by modulating the ferroptosis pathway [22]. To investigate whether GRb1 exerts its antidepressant effects through the ferroptosis pathway, we administered GRb1 intragastrically to mice after 7 days of exposure to CRS. The treatment continued for 21 days, while the control group received saline intragastrically. Behavioral tests revealed that GRb1-treated mice exhibited an increased preference for sucrose water and reduced immobility times in the FST, demonstrating GRb1′s antidepressant effect in the CRS-induced depression model (Figure 5A,B). Then, we analyzed the FTO and BECN1 gene expression levels in the hippocampus of GRb1-treated mice. Compared with the saline-treated group, the GRb1-treated group exhibited significantly increased mRNA expression of both Fto and Becn1 (Figure 5C,D). Similarly, protein expression analysis revealed that the levels of FTO, BECN1, xCT, and GPX4 were significantly increased in the GRb1-treated group compared with the control group (Figure 5E–I). Additionally, we measured the GSH and MDA levels in the hippocampus of GRb1-treated mice. The results revealed a significant increase in GSH levels and a significant decrease in MDA levels in the GRb1-treated group compared with the saline-treated group (Figure 5J,K). Our findings suggest that GRb1 may inhibit ferroptosis by upregulating the demethyltransferase FTO, thus mediating its antidepressant effect.

## 3. Discussion

In this study, we investigated the role of epigenetics in ferroptosis using cadaveric brains from MDD patients and a CRS-induced mouse model of depression. Our findings revealed that the hippocampal expression levels of FTO and BECN1 were significantly reduced in MDD patients. BECN1, a target of FTO, plays a key role in ferroptosis regulation. FTO deficiency in the hippocampus promotes ferroptosis by increasing m6A methylation on Becn1 mRNA, leading to decreased BECN1 protein levels. Conversely, FTO-OE reduces m6A methylation on Becn1 mRNA, enhancing BECN1 protein levels and inhibiting ferroptosis. Importantly, GRb1 treatment inhibited ferroptosis via the system Xc^−^-GSH-GPX4 pathway, contributing to its antidepressant effects. These findings highlight the interplay between epigenetics and ferroptosis in depression, offering potential therapeutic avenues for treating MDD.

MDD is a prevalent and severe mental health condition, characterized by persistent low mood and loss of interest, posing significant challenges to personal well-being and public health. With the rising incidence of depression, understanding its underlying mechanisms has become increasingly critical. Recent research highlights strong links between the pathogenesis of depression and both ferroptosis dysregulation [29,30] and epigenetic abnormalities [31,32,33]. In this study, using cadaveric brain samples from MDD patients and a CRS-induced depression mouse model, we identified a key role for the RNA demethyltransferase FTO in regulating ferroptosis. FTO modulates the m6A methylation level of Becn1 mRNA, which in turn influences BECN1 protein expression. These findings provide novel insights into the interplay between epigenetics and ferroptosis in the pathology of MDD.

BECN1 is a critical regulator of autophagy within cells [34]. Studies have shown that BECN1 contributes to ferroptosis through both autophagy-dependent and autophagy-independent mechanisms. For example, in mouse xenograft models, the BECN1-activating peptide Tat-beclin 1 has been demonstrated to induce autophagy-dependent ferroptosis [35]. Additionally, in cancer cells, BECN1 promotes ferroptosis by directly interacting with xCT, thereby inhibiting system Xc^−^ activity [36]. Consistent with these findings, our study revealed that BECN1 regulates xCT expression. In particular, knocking down BECN1 in cells significantly reduced xCT protein expression, further emphasizing the role of BECN1 in regulating ferroptosis.

Ferroptosis, an iron-dependent form of programmed cell death, is critically regulated by the biosynthesis of GSH, which plays an essential role in protecting cells from oxidative stress [37]. Under normal conditions, the cellular antioxidant system prevents the initiation of ferroptosis. However, disruption of this system, particularly the inactivation of the system Xc^−^-GSH-GPX4 antioxidant pathway, leads to lipid peroxide accumulation and induces ferroptosis. Our study demonstrated that in the hippocampus of CRS model mice, the antioxidant system was significantly disrupted. Specifically, the protein expression levels of xCT and GPX4 were decreased, accompanied by a reduction in GSH synthesis. These findings highlight an imbalance in the antioxidant defense system, which contributes to the induction of ferroptosis in the hippocampi of CRS model mice.

GRb1, the primary active compound extracted from ginseng roots, exhibits significant antidepressant effects in rodent models of stress-induced depression. GRb1 is known to inhibit astrocyte pyroptosis via the mitophagy and NF-κB pathways, thereby reducing inflammation and enhancing synaptic plasticity [20]. Additionally, GRb1 increases brain-derived neurotrophic factor (BDNF) levels in the hippocampus and activates the AKT signaling pathway, and these phenomena further contribute to the antidepressant effects of GRb1 [38]. Our study also demonstrated that GRb1 exerts its antidepressant effects by regulating the antioxidant system involved in ferroptosis through RNA methylation. In CRS-induced depression mice that were treated with GRb1, hippocampal FTO expression levels were increased significantly. FTO reduced the m6A methylation of Becn1 mRNA, leading to increased BECN1 protein expression. This upregulation of BECN1 stabilized the system Xc^−^-GSH-GPX4 antioxidant pathway, thereby inhibiting ferroptosis. Although chronic diseases such as neurological disorders, cardiovascular diseases, and diabetes present with different clinical manifestations, they share common features, including chronic inflammation [39,40], oxidative stress [41], and lipid abnormalities [42,43,44]. Our findings may serve as a foundation for developing future therapeutic strategies for similar chronic conditions.

The main limitation of this study is that, although we identified FTO as a regulator of the m6A modification level of Becn1 mRNA, we did not pinpoint the exact base being modified. Additionally, increasing the sample size could provide more robust evidence to evaluate whether BECN1 can serve as a potential target for depression treatment. Building on our current research, we aim to precisely identify the specific m6A modification sites on BECN1 mRNA and develop gene-targeted therapeutic strategies. Furthermore, we seek to explore the potential of ferroptosis-related markers as diagnostic and prognostic biomarkers for MDD.

## 4. Materials and Methods

### 4.1. Cadaveric Brain Samples

Human hippocampal tissues were obtained from the Human Brain Bank, Chinese Academy of Medical Sciences & Peking Union Medical College, Beijing, China. The human tissues were donated voluntarily and free of charge, and informed consent was provided by the donor and all immediate family members. Donated tissues could be used only for scientific research purposes. The autopsy interval between donor death and brain tissue collection was less than 40 h, and detailed clinical information of the donors is provided in the attached table. Further details are provided in Table 1.

#### Sample Processing

According to the Human Brain Bank guidelines, the hippocampus is divided into ten segments. We selected the 5th or 6th segment, which represents the middle region of the hippocampus. The selected sample was placed in PBS, and after the addition of steel beads, it was homogenized using a tissue grinder. The resulting homogenate was then used for subsequent experimental analyses.

### 4.2. Animals

Four-week-old male C57BL/6J mice were obtained from Weitonglihua Animal Technology Co., Ltd. (Beijing, China). The mice were maintained under standard housing conditions, including a 12 h light/dark cycle and a controlled temperature of 25 ± 2 °C, for four weeks prior to experimental use. All the animal experiments complied with the institutional guidelines set by the Beijing Administration Office of Laboratory.

### 4.3. Cell Culture

The 293T cell line was obtained from the Cell Resource Center (IBMS, London, UK, CAMS/PUMC) and cultured in DMEM supplemented with 10% irradiated fetal bovine serum, 100 IU/mL penicillin, and 0.1 mg/mL streptomycin. The cells were maintained at 37 °C in a humidified atmosphere with 5% CO_2_. Becn1-siRNA was transfected into 293T cells using Lipofectamine™ RNAiMAX (13778150, Thermo, Waltham, MA, USA) according to the manufacturer’s instructions.

### 4.4. Drug Administration

Ginsenoside Rb1 (HY-N0039, MedChemExpress, Monmouth Junction, NJ, USA) was diluted in 0.9% saline to prepare a 2 mg/mL solution. Starting on day 8 of CRS modeling, GRb1 was administered by gavage at a dose of 20 mg/kg daily for 21 days. The control group received 0.9% saline under identical conditions, including the same concentration, method of administration, and duration.

### 4.5. Vector Construction and rAAV Packaging

Human-si-Becn1 (SKU: siB160225095753-1-5) was obtained from RiboBio (RiboBio Co., Ltd., Guangzhou, China) at a concentration of 5 nmol. rAAV vectors related to FTO were constructed and packaged by Taitool Bioscience (https://www.taitool.com/index.jsp, Taitool Bioscience Co., Ltd., Shanghai, China). The rAAV vectors that were used in this study included the following: AAV-CAG-Fto-2A-EGFP (Cat. No.: AAV2/9-XT071), AAV-U6-Fto shRNA-CMV-ZsGreen (Cat. No.: AAV2/9-XT071), and AAV-CMV-bGlobin-eGFP-WPRE (Cat. No.: AAV2/9-S0263-9-H50). The final titer of each rAAV preparation ranged from 3 × 10^12^ to 4 × 10^12^ vector genomes (v.g.)/mL.

### 4.6. CRS Model Establishment

To establish the CRS model, 50 mL centrifuge tubes were modified by punching small holes in their surfaces and bottoms to ensure adequate air circulation. We established four experimental groups, namely, the control group, CRS treatment group, CRS treatment + saline group, and CRS treatment + GRb1 group. Mice were placed individually into the centrifuge tubes for 4 h daily, during which they had no access to food or water. After the restraint period, the mice were returned to their original cages. This procedure was carried out for 28 consecutive days, with the timing of stress exposure varying randomly each day to introduce unpredictability in the stress stimulation.

### 4.7. Behavioral Testing Protocol

The mice were acclimated to the testing environment 1 h before each behavioral test. The testing environment was maintained under conditions of quietness, sound insulation, and low-light illumination to minimize external stressors.

#### 4.7.1. FST

The FST was conducted using a 2 L beaker filled with water at a depth sufficient to prevent the mice from touching the bottom while also ensuring that they could remain afloat without sinking. The water temperature was maintained between 23 and 25 °C. Each mouse was placed in the beaker for a total of six minutes, during which the entire session was recorded. The last four minutes of immobility were used for data analysis. The mice were considered immobile if they floated in the water with minimal movement, such as slight motion of the forelimbs. Data analysis was conducted using a single-blind design to ensure the statisticians were unaware of the experimental treatment, maintaining objectivity.

#### 4.7.2. SPT

The sucrose preference test included a 72 h adaptation period followed by the testing phase. During the adaptation period, ordinary water and a 1.5% sucrose solution were provided at designated positions in the mouse cage. At the start of the adaptation period (0 h), the initial placements of water and sucrose solution bottles were set. At 24 h, the positions of the two bottles were swapped to eliminate positional bias. At 48 h, water was removed, and the mice were deprived of water for 24 h, although food remained available.

The testing phase began immediately after the adaptation period. The initial weights of the bottles were recorded at 0 min (start of the test). Both water and sucrose solution were placed in their respective positions. After 1 h, the positions of the bottles were swapped to avoid positional preference. The final weights of the bottles were recorded at 2 h. The sucrose preference ratio, calculated as the ratio of consumed sucrose solution to total liquid intake, was used as an indicator of anhedonia in the mice.

### 4.8. cDNA Synthesis

Total RNA was extracted from the hippocampus using TRIzol reagent, and RNA concentrations were measured with a NanoDrop spectrophotometer. 1 µg of total RNA was reverse-transcribed into cDNA using the PrimeScript™ RT Reagent Kit with gDNA Eraser (RR047A, TakaRa, Shiga-ken, Japan), which also removes genomic DNA contamination during the process.

### 4.9. RT-qPCR

Quantitative PCR was performed using the LightCycler 96 Real-Time System (05815916001, Roche, Basel, Switzerland) with FastStart Essential DNA Green Master Mix (06924204001, Roche, Basel, Switzerland). Each sample was analyzed with three technical replicates. Gapdh was amplified and used as the internal reference gene. Differences in gene expression were calculated using the 2^−ΔΔCT^ method.

### 4.10. MeRIP-qPCR

To isolate mRNA in the hippocampus of FTO EGFP, KD, and OE mice, total RNA was purified using Dynabeads™ Oligo(dT)25 (61002, Thermo). A minimum of 5 µg of mRNA is required for each sample. m6A immunoprecipitation was performed following the protocol provided with the Magna MeRIP m6A Kit (17-10499, Merck, Darmstadt, Germany). RT-qPCR was conducted using the HiScript II One Step qRT-PCR SYBR Green Kit (Q221, Vazyme, Nanjing, China), with RNA as the template. The relative expression of m6A-modified genes was calculated as the ratio of the CT value of m6A IP to the CT value of Input. For primer information, please refer to Table 2.

### 4.11. Ferroptosis PCR Array

Synthesized cDNA was mixed with FastStart Essential DNA Green Master Mix (06924204001, Roche) and applied to a Ferroptosis PCR Array 96-well plate (wc-mRNA0271-H, WcGene Biotech, Shanghai, China) for RT-qPCR. Actb was used as the internal reference gene. Gene expression levels were determined using the same 2^−ΔΔCT^ method as described for the RT-qPCR analysis.

### 4.12. Stereotaxic Surgery on Mouse Brains

Eight-week-old mice were anesthetized via intraperitoneal injection of 0.7% pentobarbital. rAAV was stereotaxically injected into the bilateral hippocampus using the following coordinates relative to the bregma: 2.2 mm posterior, ±2.2 mm lateral, and 1.8 mm ventral. The injections were performed with a Microsyringe pump controller (Micro4, World Precision Instruments, Sarasota, FL, USA) at a dose of 1 µL per site and a rate of 300 nL/min. The expression of rAAV genes was assessed 4 weeks after stereotaxic surgery to confirm the effectiveness of the injection.

### 4.13. Western Blot Analysis

Brain samples were lysed with RIPA buffer supplemented with a protease inhibitor cocktail. The samples were placed in a steel bead and homogenized using a fully automatic sample fast grinder. After homogenization, the samples were subjected to ultrasonic disruption and centrifugation. Protein quantification was performed using the Pierce BCA Protein Assay Kit (23225, Thermo). After quantification, the samples were mixed with 5X Omni-Easy™ Protein Sample Loading Buffer and denatured at room temperature for 30 min. A total of 20 µg of protein per sample was separated using 12% sodium dodecyl sulfate-polyacrylamide gel electrophoresis (SDS-PAGE) and transferred onto nitrocellulose membranes. The membranes were blocked using Fast Blocking Western solution and incubated at room temperature for 20 min, followed by overnight incubation with primary antibodies at 4 °C. The primary antibodies used were anti-FTO (ab92821, abcam, Cambridge, UK), anti-BECN1 (ab207612, abcam, Cambridge, UK), anti-xCT (ab307601, abcam, Cambridge, UK), anti-GPX4 (67761-1-lg, proteintech, Wuhan, China), and anti-β-actin (20536-1-AP, proteintech, Wuhan, China). The membranes were then incubated at room temperature for 1 h with goat anti-rabbit IgG/HRP (SE134, Solarbio, Beijing, China) and goat anti-mouse IgG/HRP (SE131, Solarbio, Beijing, China) as secondary antibodies. Protein bands were visualized with enhanced chemiluminescence (ECL) reagent, and the results were recorded.

### 4.14. Enzyme-Linked Immunosorbent Assay (ELISA) Analysis

Hippocampal tissues were isolated, minced into small pieces, and homogenized in PBS using a fully automatic sample rapid grinder with steel beads. After homogenization, the samples were ultrasonicated and centrifuged to prepare the supernatants for analysis. The GSH ELISA Kit (D75008, Sangon Biotech, Shanghai, China) and MDA ELISA Kit (D751053, Sangon Biotech, Shanghai, China) were used to measure glutathione (GSH) and malondialdehyde (MDA) levels, respectively, following the manufacturers’ instructions. The concentrations of GSH and MDA were inversely proportional to the optical density (OD) values obtained. A standard curve was generated, and concentrations were calculated using the four-parameter logistic (4PL) model for data analysis.

### 4.15. Statistical Analysis

Statistical analysis was conducted using Prism software (10.3.0). A *p* value < 0.05 was considered to indicate statistical significance. Data are presented as the means ± standard errors of the means (s.e.m.).

## Figures and Tables

**Table 1:** Information of postmortem human brains.

	Sex	Age (Years)	Autopsy Delay (h)
Control	2 females 1 male	81–87	6–17.6
Depression	2 females 1 male	72–87	6–39.0

**Table 2:** Primers for qRT-PCR used in the experiments.

Gene	Forward Sequences	Reverse Sequences
mBecn1	5′-ACAGCTCCATTACTTACCAC-3′	5′-GTAGACATCATCCTGGCTG-3′
mFto	5′-CTATAGCTGCGAAGGCTCTG-3′	5′-TAGCAGTCTCCCTGGTGAAGA-3′
mGapdh	5′-TGTGTCCGTCGTGGATCTGA-3′	5′-TTGCTGTTGAAGTCGCAGGAG-3′
Becn1 Gene-specific m6A qPCR	5′-GGACGTGGAGAAAGGCAAGA-3′	5′-CGTCAGCATGAACTTGAGCG-3′

## Data Availability

The data used in the current study are available from the corresponding author upon reasonable request.
